# Supplementary material for: Bio-Skin-Inspired Flexible Pressure Sensor Based on Carbonized Cotton Fabric for Human Activity Monitoring
Source: Sensors (Basel). 2024 Jul 3;24(13):4321. doi: 10.3390/s24134321 (PMC11243851; doi:10.3390/s24134321)
Supplement: Supplementary file 1 [file sensors-24-04321-s001.zip › sensors-3044542-supplementary.pdf]

Supplementary Materials

Bio-Skin-Inspired Flexible Pressure Sensor Based on Carbonized Cotton Fabric for Human Activity Monitoring

Min Yang [1], Zhiwei Wang [2], Qihan Jia [2], Junjie Xiong [3,*] and Haibo Wang [2,*]

[1] Division of Oncology, Department of Paediatric Surgery, West China Hospital of Sichuan University, Chengdu 610041, China; hx2014bsym@163.com

[2] College of Biomass Science and Engineering, Sichuan University, Chengdu 610065, China; zhiweiwang898@gmail.com (Z.W.); jiaqihan@stu.scu.edu.cn (Q.J.)

[3] Division of Pancreatic Surgery, Department of General Surgery, West China Hospital, Sichuan University, Chengdu 610041, China

* Correspondence: junjiex2011@126.com (J.X.); whb6985@scu.edu.cn (H.W.); Tel.: +86-28-85401296 (H.W.)

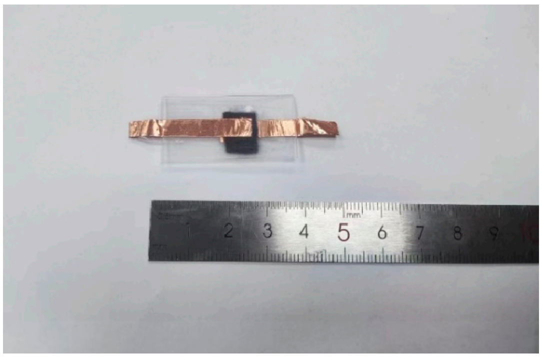

Figure S1. The optical photo of the skin-like MFPS device

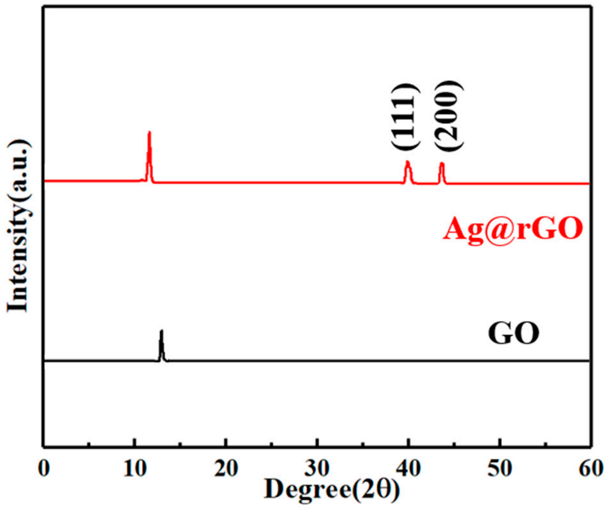

Figure S2. The XRD patterns of GO and Ag@rGO.

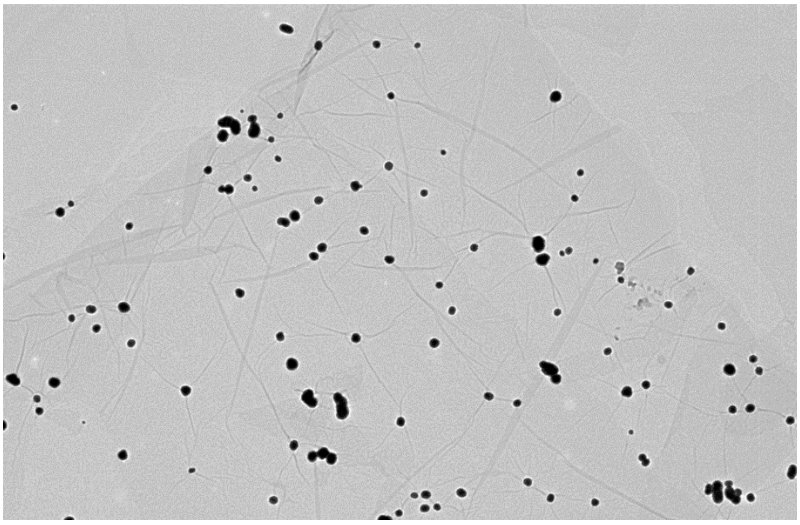

Figure S3. The high-resolution image of Ag@rGO.

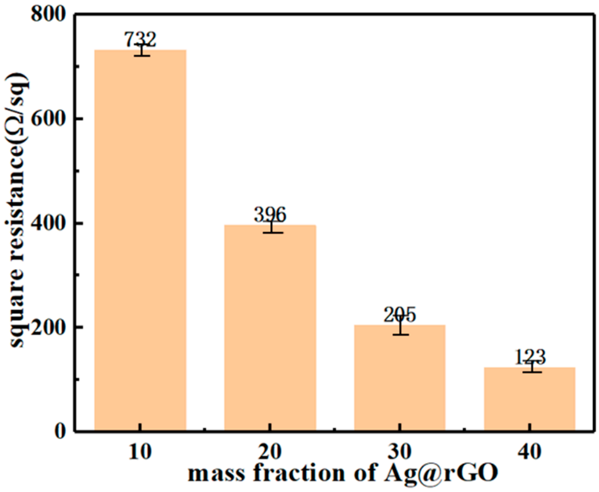

Figure S4. The square resistance values of different mass-fraction Ag@rGO in PMDS.

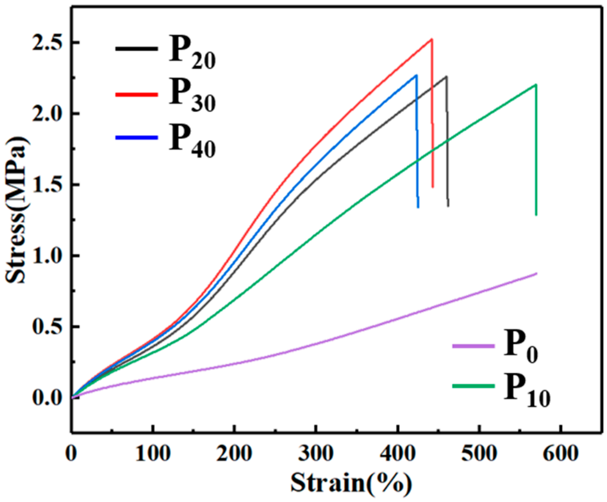

Figure S5. Mechanical properties of different mass-fraction Ag@rGO in PMDS (P_x: x represents Ag@rGO content).

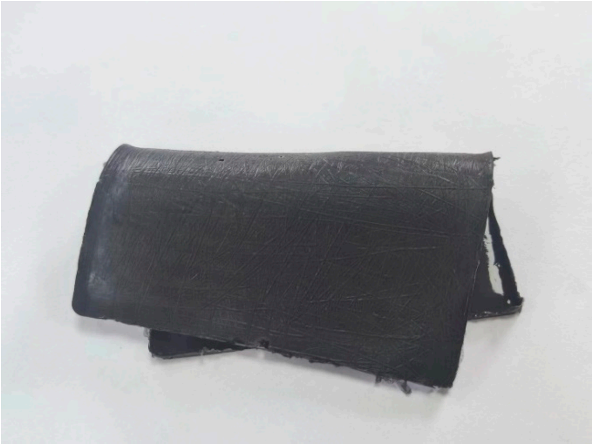

Figure S6. High resolution image with wrinkled structured electrodes

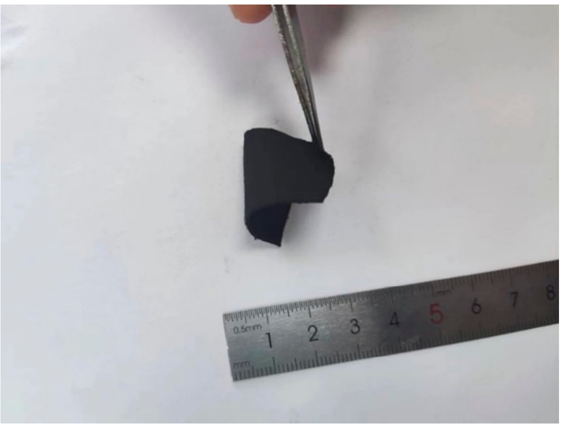

Figure S7. Optical diagram of curved carbonized fabric.

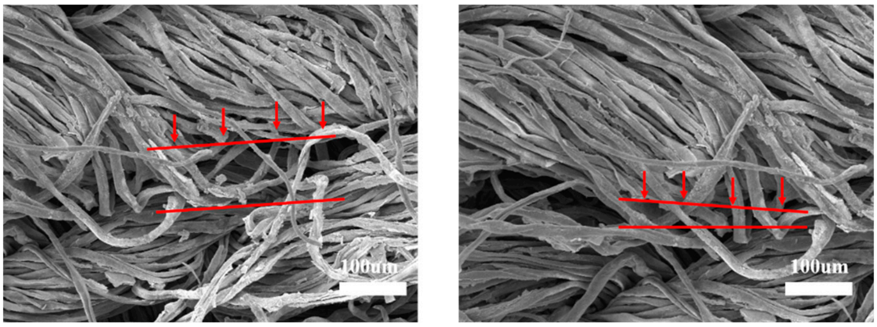

Figure S8. Schematic diagram of sensing mechanism

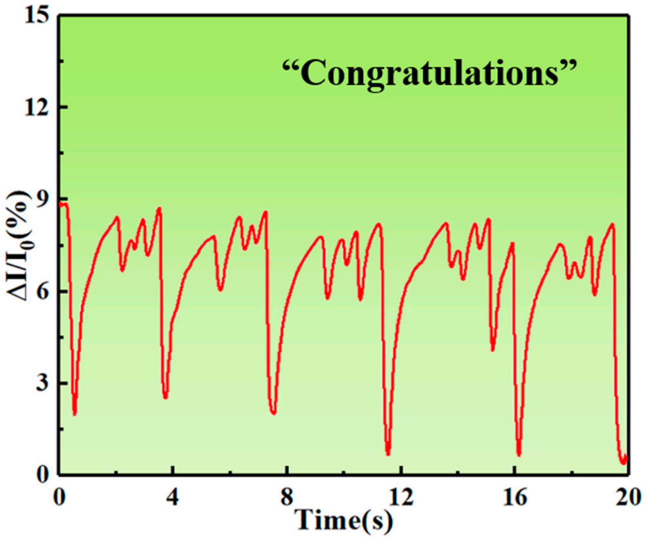

Figure S9. Current change of sensor when speaking "congratulations".

Table S1 Performance comparison of this work with previous related studies

Materials	Methods	Sensitivity(kPa^{-1})	Range(kPa)	Response time(ms)	Ref.
Ti3C2Tx@nonwoven Fabric	dip-coating	6.31	0-150	300/260	[1]
graphene/gelatin	dip–drying	1.08	0-15	60/N.	[2]
MXene/cotton fabric	dip-coating	2.27	0-150	50/20	[3]
cellulose fabric/rGO	carbonizing	2.77	0-12	40/80	[4]

PEDOT/polyester nonwoven fabric	Situ vapor phase polymerization	5.42	0-40	60/60	[5]
CNT/TPU	3D printing	1.02	0-160	N./N.	[6]
Ag@rGO/carbonized fabric/PDMS	Carbonizing and assembling	5.51	0-200	60/90	This work

References:

[1]Q. Yu, C. Su, S. Bi, Y. Huang, J. Li, H. Shao, J. Jiang and N. Chen, *ACS Appl. Mater. Interfaces* **2022**, *14*, 9632-9643.

[2]Y. Zhao, L. Liu, Z. Li, F. Wang, X. Chen, J. Liu, C. Song and J. Yao, *J. Mater. Chem. C* **2021**, *9*, 12605-12614.

[3]Y. Zheng, R. Yin, Y. Zhao, H. Liu, D. Zhang, X. Shi, B. Zhang, C. Liu and C. Shen, *Chem. Eng. J.* **2021**, *420*, 127720.

[4]R. Wu, L. Ma, A. Patil, Z. Meng, S. Liu, C. Hou, Y. Zhang, W. Yu, W. Guo and X. Y. Liu, *J. Mater. Chem. A* **2020**, *8*, 12665-12673.

[5]J.-w. Zhang, Y. Zhang, Y.-y. Li, X. Ye, P. Wang and Y. Xu, *ACS Appl. Electr. Mater.* **2021**, *3*, 3177-3184.

[6]Y. M. Yin, H. Y. Li, J. Xu, C. Zhang, F. Liang, X. Li, Y. Jiang, J. W. Cao, H. F. Feng, J. N. Mao, L. Qin, Y. F. Kang and G. Zhu, *ACS Appl. Mater. Interfaces* **2021**, *13*, 10388-10396.
